# Supplementary material for: Evaluation of the peer leadership for physical literacy intervention: A cluster randomized controlled trial
Source: PLoS One. 2023 Feb 16;18(2):e0280261. doi: 10.1371/journal.pone.0280261 (PMC9934439; doi:10.1371/journal.pone.0280261)
Supplement: S1 Fig — (DOCX) [file pone.0280261.s001.docx]

| **Figure 1. SPIRIT Diagram** | | | | |
| --- | --- | --- | --- | --- |
|  | **STUDY PERIOD** | | | |
|  | **Enrolment** | **Allocation** | **Post-allocation**  **(weeks)** | **Close-out** |
| **TIMEPOINT**** | ***-t_1_*** | **0** | **24** | ***t_x_*** |
| ENROLMENT: |  |  |  |  |
| Eligibility screen | X |  |  |  |
| Informed consent | X |  |  |  |
| *Informed assent* | X |  |  |  |
| Allocation |  | X |  |  |
| **INTERVENTIONS:** |  |  |  |  |
| *Peer Leaders for Physical Literacy* |  |  |  |  |
| *Waitlist-Control ‘usual practice’ condition* |  |  |  |  |
| **ASSESSMENTS:** | | | | |
| ***Primary Outcome*** | | | | |
| *Teacher Rated Transformational Leadership* | x |  | x |  |
| ***Secondary Outcomes*** | | | | |
| *Grade 6/7 Student-Rated Transformational Leadership* | x |  | x |  |
| *Grade 6/7 Leadership Self-Efficacy* | x |  | x |  |
| *Grade 6/7 Post-Test Program Evaluation* |  |  | x |  |
| *Grade 3/4 Self-Determined Motivation* | x |  | x |  |
| *Grade 3/4 Perceived Competence* | x |  | x |  |
| *Grade 3/4 Self-Concept* | x |  | x |  |
| *Grade 3/4 Movement Skill Competence* | x |  | x |  |
| *Grade 3/4 Physical Activity* | x |  | x |  |
| *Program Adherence* |  |  | x |  |
