# Supplementary material for: Evaluation of the peer leadership for physical literacy intervention: A cluster randomized controlled trial
Source: PLoS One. 2023 Feb 16;18(2):e0280261. doi: 10.1371/journal.pone.0280261 (PMC9934439; doi:10.1371/journal.pone.0280261)
Supplement: S2 Table — (DOCX) [file pone.0280261.s003.docx]

| **Supplementary Table 2. Intervention Lessons, Goals, and Exemplar Activities** | | |
| --- | --- | --- |
| **Lesson** | **Goal** | **Exemplar Activities** |
| Lesson #1  (Location: classroom) | Introduction | - Peer leaders are provided with a leader handbook (to reinforce positive leadership qualities and remind them of each of the movement skills, key teaching points, and examples of warm-up, skill activities, and small sided games to play with their groups)  and a whistle. Grade 6/7 teachers are also provided with additional laminated lesson plans (stored in the class).  - Experience elicitation activity (using diverse examples of leadership from sports, popular culture, politics, and history).  - Good versus bad leader game. Students asked to think of the best and worst leaders they have had, and identify behaviors/qualities that defined them as good or bad, and consider how those good versus bad leaders made students feel).  - Provide students with a working definition of exceptional leadership (emphasizing the importance of “the small things we do having a big impact”, and “leadership is about lifting others up”). |
| Lesson #2  (Location: classroom) | Cover ‘role modeling’ and ‘motivating others’ | - Convey the importance of leadership behaviors in school and beyond (e.g., work, families, sport)  - Use film footage to convey ‘key’ ideas and stimulate discussion (in small groups and as a class). For example, key excerpts from Nelson Mandela’s life are used (e.g., using footage from the 16^th^ Man, (Bestall, 2010), as well as documentary-style film footage (titled ‘Role Modeling’ and ‘Motivating Others’, Lantern Films) created specifically for the PLPL intervention to explain/illustrate how 12 year old students can display role modeling and motivate other students.  - Question and answer activities. Questions directly related to the film footage are used (e.g., “Why did Nelson Mandela wear a Springbok jersey and hat”?), as well as bridging questions to make relevant this footage to their forthcoming roles as peer leaders (e.g., “what are some of the ‘small’ things you can do to positively influence others”).  - As a complement to small group idea elicitation discussions and activities, provide students with a short list of exemplar ‘behaviors’ that reflect each leadership dimension (didactic delivery). Discuss each behavior. |
| Lesson #3  (Location: classroom) | Cover ‘considering others’ and ‘helping others to think’ | - Thought experiment – ask whether students can remember the name of their Grade 6 or 7 ‘buddy’ from when they were in kindergarten or Grade 1. Emphasize the long-lasting effects of (good and bad) leadership.  - Show short film ‘considering others’ and ask students (in small groups) what they think ‘being considerate of others involves. Use idea elicitation to identify tangible behaviors. Juxtapose these against list of evidence-informed established (individualized consideration) behaviors. - Thought experiment (taken from Clive Woodward’s book ‘Winning’, (Woodward, 2005), with students asked to count the number of Fs in “Finished files are the result of years of scientific study combined with the experience of many years.” This activity is used to illustrate the idea of addressing current challenges/problems in new ways (hint: the answer to the stimulus question in ‘six fs’). - Role play activity (based on movement skill acquisition) to illustrate how peer leaders can help younger students to think about challenges in new ways. To illustrate, when asked “what part of my foot should I kick with?”) a peer-leader’s response could involve providing the answer (i.e., typical response) or encouraging the student to think for him or herself (e.g., “what do think would work best (and why?)?”). |
| Lesson #4  (Location: classroom) | Introduction to movement skills | - Short film (entitled ‘Movement Skills’), using humour highlights the benefits of movement skills, as well as the six movement skills (i.e., kick, catch, two-handed batting, dribble, underarm throw, and overarm throw) peer leaders will subsequently be teaching in the 10 week movement skills program.  - Provide slides that highlight the ‘key’ teaching points related to each of the six movement skills. Encourage peer leaders to learn those key teaching points and subsequently check for understanding (via homework and quiz).  - Explain (via didactic delivery) how peer leaders will use their leadership skills to deliver a 10-week movement skills program to Grade 3/4 students. Workshop facilitators will:  - explain activity session structure (i.e., warm-up, demonstrate targeted skill, skill practice, skill application to small-sided game, cool down)  - explain schedule/timetable for activity sessions embedded in the 10-week program)  - work through exemplar lesson plans (laminated lesson plans are provided for peer-leaders to successfully teach each movement skill), and provide an overview of ‘key points’ to running an activity session.  - PSA style video on ‘classroom management’: emphasis on key points for (a) general classroom management, and (b) managing disruptive students  - Review the games and activities (provided in the peer leader handbook) for understanding.  - Overview of feedback and support that peer leaders will receive from peers, their teacher, and from the research team. |
| Lesson #5  (Location: Gymnasium) | Experience demonstrating movement skills | - Peer leaders provided the opportunity to teach each other the six movement skills and obtain feedback from each other.  - After setting up the gymnasium and conducting a warm-up (led by class teacher), students work in groups of three (at six skill ‘stations’). One student demonstrates the skills (e.g., overarm throw), with the second receiving/retrieving the ball, and the third checking that the correct teaching points are delivered (using the student handbook) and providing feedback to the demonstrator. Individualized feedback is also provided by the class teacher. After 5 mins rotate stations.  - Finish with a game (selected from the peer leader handbook), for 10 mins (led by class teacher), and explain that peer leaders will be responsible for the game for the next class.  Homework: Peer leaders familiarize themselves with each practice activity and game in the leadership handbook. Follow up with in-class discussion to check understanding of activities, skill practices, and games. |
| Lessons #6-7  (Location: Gymnasium) | Peer Leader Teaching Practice | - Peer leaders work in pairs to teach one section of an activity session (e.g., warm-up, demonstration, skill practice, game). It is critical that students plan their activity (e.g., warm-up) in advance of the actual session taking place (teachers are encouraged to build in planning time before this class).  - To maximize opportunities for peer leaders to accrue teaching practice, split class into two and have students run parallel classes within the same gym space. Each pair of students should have the opportunity to practice teaching at least two sections of an activity session. |
