# Supplementary material for: Evaluation of the peer leadership for physical literacy intervention: A cluster randomized controlled trial
Source: PLoS One. 2023 Feb 16;18(2):e0280261. doi: 10.1371/journal.pone.0280261 (PMC9934439; doi:10.1371/journal.pone.0280261)
Supplement: S3 Table — (DOCX) [file pone.0280261.s004.docx]

| **Supplemental Table 3.** Teacher and Grade 6/7 Student Outcomes Cronbach Alpha, ICC, and Design Effects | | | | | | |
| --- | --- | --- | --- | --- | --- | --- |
|  | **Baseline** | | | **Follow-Up** | | |
| **Outcome** | **α** | ***ICC*** | **Design Effect** | **α** | ***ICC*** | **Design Effect** |
| Teacher-Rated Transformational Leadership | 0.919 | 0.225 | 5.015 | 0.929 | 0.174 | 4.103 |
| Student-Rated Transformational Leadership | 0.653 | 0.191 | 4.414 | 0.703 | 0.142 | 3.531 |
| Leadership Self-Efficacy | 0.930 | 0.196 | 4.496 | 0.932 | 0.207 | 4.700 |
| ICC = Intraclass correlation coefficient | | | | | | |
