# Supplementary material for: Evaluation of the peer leadership for physical literacy intervention: A cluster randomized controlled trial
Source: PLoS One. 2023 Feb 16;18(2):e0280261. doi: 10.1371/journal.pone.0280261 (PMC9934439; doi:10.1371/journal.pone.0280261)
Supplement: S4 Table — (DOCX) [file pone.0280261.s005.docx]

| **Supplemental Table 4.** Grade 3/4 Student Outcomes Cronbach Alpha, ICC, and Design Effects | | | | | | |
| --- | --- | --- | --- | --- | --- | --- |
|  | **Baseline** | | | **Follow-Up** | | |
| **Outcome** | **α** | ***ICC*** | **Design Effect** | **α** | ***ICC*** | **Design Effect** |
| Self-Determined Motivation | 0.858 | 0.004 | 1.061 | 0.894 | 0.041 | 1.631 |
| Perceived Competence | 0.836 | 0.007 | 1.110 | 0.874 | 0.028 | 1.433 |
| Self-Concept | 0.932 | 0.067 | 2.014 | 0.947 | 0.046 | 1.705 |
| Movement Skill Competence |  |  |  |  |  |  |
| Maximal Throw Speed |  | 0.072 | 2.091 |  | 0.039 | 1.593 |
| Maximal Kick Speed |  | 0.087 | 2.326 |  | 0.127 | 2.938 |
| Throw Components |  | 0.054 | 1.826 |  | 0.019 | 1.285 |
| Kick Components |  | 0.142 | 3.16 |  | 0.232 | 4.528 |
| Throw-Catch Combo |  | 0.069 | 2.048 |  | 0.061 | 1.934 |
| School Day MVPA |  | 0.023 | 1.068 |  | 0.121 | 1.363 |
| ICC = Intraclass correlation coefficient | | | | | | |
