# Supplementary material for: Evaluation of the peer leadership for physical literacy intervention: A cluster randomized controlled trial
Source: PLoS One. 2023 Feb 16;18(2):e0280261. doi: 10.1371/journal.pone.0280261 (PMC9934439; doi:10.1371/journal.pone.0280261)
Supplement: S5 Table — (DOCX) [file pone.0280261.s006.docx]

| **Supplemental Table 5.** Peer Leader Program Evaluation | |
| --- | --- |
| **Component Evaluated** | **Program Evaluation Score**  n = 74  ***Mean (Standard Deviation)*** |
| I enjoyed participating in the PLPL program | 2.47 (0.58) |
| The PLPL program helped me to be a better leader | 2.54 (0.60) |
| I enjoyed the PLPL leadership training | 2.81 (0.68) |
| I found the student manual useful for the PLPL sessions | 2.53 (0.69) |
| The students in my group enjoyed the PLPL sessions | 2.66 (0.71) |
| I enjoyed the length of each PLPL session | 2.74 (0.66) |
| As a PLPL leader, I found the feedback given to me to be useful | 2.46 (0.71) |
| I found the lesson cards useful for the PLPL sessions | 2.55 (0.71) |
| I found the equipment kits included enough equipment | 2.43 (0.66) |
| I enjoyed being a PLPL leader | 2.39 (0.59) |
