# Supplementary material for: Evaluation of the peer leadership for physical literacy intervention: A cluster randomized controlled trial
Source: PLoS One. 2023 Feb 16;18(2):e0280261. doi: 10.1371/journal.pone.0280261 (PMC9934439; doi:10.1371/journal.pone.0280261)
Supplement: S1 File — (DOCX) [file pone.0280261.s008.docx]

**Teacher Peer Leadership Questionnaire**

**Teacher name: __________________________**

**Teacher school: _________________________**

**Date (MM/DD/YYYY):___________________**

In this section, we would like you to describe the leadership style of the student peer leader that you are rating/observing. To answer each question, please circle the number that best describes what you think. **If a question is irrelevant, or if you are unsure or do not know the answer, leave the answer blank.** Please be as honest as possible, and answer how frequently each statement fits the student you are describing.

*Use the following rating scale:*

| **Not at all** | **Once in a while** | **Sometimes** | **Fairly often** | **Frequently** |
| --- | --- | --- | --- | --- |
| 0 | 1 | 2 | 3 | 4 |

***The peer leader that I’m rating is _________________________ (student name)***

| Shows that s/he cares about the students s/he is teaching | 0 | 1 | 2 | 3 | 4 |
| --- | --- | --- | --- | --- | --- |
| Is enthusiastic about what other students are capable of achieving | 0 | 1 | 2 | 3 | 4 |
| Encourages students to think for themselves | 0 | 1 | 2 | 3 | 4 |
| Behaves as someone that other students can trust | 0 | 1 | 2 | 3 | 4 |

|  |  |
| --- | --- |

**FOR OFFICE USE ONLY- P/ I.D.**

Peer Leadership for Physical Literacy (PLPL) Study

Grade 6/7 students

Leadership Questionnaire

***ID:****____________________*

***Teacher’s name:****____________________*

***School Name:****_______________________*

***What is today’s date:*** *_______ (Day) _______ (Month) 20______ (Year)*

**Background information**

**PART A: Background Information**

***A1.*** *Date of Birth: _______ (Day)_______(Month) 20_______(Year)*

***A2.*** *Where were you born:_______________(City)_________________(Country)*

***A3.*** *What is your age (years):________________*

***A4.*** *Gender (check one): Male  Female *

***A5*.** How do you describe yourself in terms of your ethnic origin? PLEASE CHECK **ALL** THAT

APPLY.

| *Canadian* |  | *East Indian* |  | *American (USA)* |  |
| --- | --- | --- | --- | --- | --- |
| *Native/Aboriginal* |  | *Dutch* |  | *Norwegian* |  |
| *Chinese* |  | *Persian* |  | *Italian* |  |
| *British* |  | *Polish* |  | *Korean* |  |
| *Irish* |  | *Hispanic* |  | *Filipino* |  |
| *German* |  | *Russian* |  | *South Asian* |  |
| *French* |  | *Vietnamese* |  | *Japanese* |  |

*Other_______________________*

***A6.*** *What is your mother/female guardian’s job?____________________*

***A7.*** *What is your father/male guardian’s job?_____________________*

**The following questions are about Leadership.**

**Part B: Peer Leadership**

In this section, we would like you to describe the your leadership style. To answer each question, please circle the number that best describes what you think. **If a question is irrelevant, or if you are unsure or do not know the answer, leave the answer blank.** Please be as honest as possible, and answer how frequently each statement fits you.

*Use the following rating scale:*

| **Not at all** | **Once in a while** | **Sometimes** | **Fairly often** | **Frequently** |
| --- | --- | --- | --- | --- |
| 0 | 1 | 2 | 3 | 4 |

| I show that I care about the students I am teaching | 0 | 1 | 2 | 3 | 4 |
| --- | --- | --- | --- | --- | --- |
| I am enthusiastic about what other students are capable of achieving | 0 | 1 | 2 | 3 | 4 |
| I encourage students to think for themselves | 0 | 1 | 2 | 3 | 4 |
| I behave as someone that other students can trust | 0 | 1 | 2 | 3 | 4 |

**PART C: *Leadership Self-confidence***

In the following questions we want you to think about how confident you would be as a Grade 6 or 7 student to teach different sports skills and physical activity games, as a peer-leader, to a small group Grade 3 or 4 students. This might involve leading a warm-up, teaching different sports skills (e.g., throwing, kicking, dribbling, catching), and then organizing games with those Grade 3/4 students.

The following questions focus on your **confidence to do different things as a peer leader in your school**. There are no right or wrong answers to any of these questions, and we would like you to **rate your confidence in your ability at this moment in time** using the following scale, where 0% means you have no confidence, 50% means you are somewhat confident and 100% means you are completely confident...

| **0%** | **10%** | | **20%** | | **30%** | **40%** | | | **50%** | **60%** | | **70%** | **80%** | | | **90%** | | **100%** |
| --- | --- | --- | --- | --- | --- | --- | --- | --- | --- | --- | --- | --- | --- | --- | --- | --- | --- | --- |
| Not at all | |  | |  | | |  | Somewhat | | |  | | |  | | | Completely | |
| **If you really wanted to, how confident are you that you can…** | | | | | | | | | | | | | | | **Confidence**  **(0-100)** | | | |
| 1. behave as someone that other students can trust | | | | | | | | | | | | | | |  | | | |
| 1. motivate other students | | | | | | | | | | | | | | |  | | | |
| 1. help other students if they are struggling | | | | | | | | | | | | | | |  | | | |
| 1. encourage students to think for themselves | | | | | | | | | | | | | | |  | | | |
| 1. lead active games and fitness activities involving other students | | | | | | | | | | | | | | |  | | | |
| 1. teach physical activity skills to other students | | | | | | | | | | | | | | |  | | | |
| 1. be enthusiastic about what other students are capable of achieving | | | | | | | | | | | | | | |  | | | |
| 1. make good decisions | | | | | | | | | | | | | | |  | | | |
| 1. help students feel safe about joining in | | | | | | | | | | | | | | |  | | | |
| 1. be a role model to other students | | | | | | | | | | | | | | |  | | | |
| 1. show that you care about the students you are teaching | | | | | | | | | | | | | | |  | | | |

**Background information**

***ID:____________________***

***Teacher’s name:____________________***

***School Name:_______________________***

***What is today’s date:*** *_______ (Day) _______ (Month) 20______ (Year)*

***A1.*** *Date of Birth: _______ (Day)_______(Month) 20_______(Year)*

***A2.*** *Where were you born:_______________(City)_________________(Country)*

***A3.*** *What is your age (years):________________*

***A4.*** *Gender (check one): Male  Female *

***A5.*** How do you describe yourself in terms of your ethnic origin? PLEASE CHECK **ALL** THAT

APPLY.* *

| *Canadian* |  | *East Indian* |  | *American (USA)* |  |
| --- | --- | --- | --- | --- | --- |
| *Native/Aboriginal* |  | *Dutch* |  | *Norwegian* |  |
| *Chinese* |  | *Persian* |  | *Italian* |  |
| *British* |  | *Polish* |  | *Korean* |  |
| *Irish* |  | *Hispanic* |  | *Filipino* |  |
| *German* |  | *Russian* |  | *South Asian* |  |
| *French* |  | *Vietnamese* |  | *Japanese* |  |

*Other_______________________*

***A6.*** *What is your mother/female guardian’s job?____________________*

***A7.*** *What is your father/male guardian’s job?_____________________*

**PART B:** **How do you feel about physical activity?**

Boys and girls can be **active** by doing all sorts of things:

- Exercise (walking, keep fit, gym)
- Playing out, doing active things (like playing in the park)
- Sports (like football, tennis, netball, swimming)

Here are some reasons why you might be active.

Please read each sentence and tell us how true it is for you (by circling the number that is most correct for you).

| **I am active because...** | | | | | | |
| --- | --- | --- | --- | --- | --- | --- |
|  |  | Not true for me | Not really true for me | Sometimes true for me | Often true for me | Very true for me |
| 1 | being active is fun | 1 | 2 | 3 | 4 | 5 |
| 2 | it is important to me to do active things | 1 | 2 | 3 | 4 | 5 |
| 3 | I enjoy being active | 1 | 2 | 3 | 4 | 5 |
| 4 | I value the benefits of being active | 1 | 2 | 3 | 4 | 5 |
| 5 | I like being active | 1 | 2 | 3 | 4 | 5 |
| 6 | in life it is important to be active | 1 | 2 | 3 | 4 | 5 |

**Part C:** **How do you feel?**

The next section has some sentences describing how people feel about BEING ACTIVE and DOING ACTIVE THINGS (like active games, playing out and doing sports).

**Please read each sentence and tell us how like you each one is.**

|  |  | Not like me at all | Not really like me | Sometimes like me | Quite a lot like me | Really like me |
| --- | --- | --- | --- | --- | --- | --- |
| 1. | When it comes to playing active games, I think I am pretty good. | 1 | 2 | 3 | 4 | 5 |
| 2. | I think I do well compared to other children | 1 | 2 | 3 | 4 | 5 |
| 3. | After working at a new activity for a while, I feel that I can do it pretty well. | 1 | 2 | 3 | 4 | 5 |
| 4. | I am happy with how good I am at doing active games. | 1 | 2 | 3 | 4 | 5 |
| 5. | When it comes to being active, I have good skills. | 1 | 2 | 3 | 4 | 5 |

Please **circle the number** which is the **most correct** statement about you.

|  | | | | | | | |
| --- | --- | --- | --- | --- | --- | --- | --- |
|  | | False True | | | | | |
|  | I feel confident when doing coordinated movements | 1 | 2 | 3 | 4 | 5 | 6 |
|  | Overall, most things I do turn out well. | 1 | 2 | 3 | 4 | 5 | 6 |
|  | Controlling movements of my body comes easily to me. | 1 | 2 | 3 | 4 | 5 | 6 |
|  | I often do exercise or activities that make me breathe hard. | 1 | 2 | 3 | 4 | 5 | 6 |
|  | I am good at most sports. | 1 | 2 | 3 | 4 | 5 | 6 |
| 6. | Physically, I am happy with myself. | 1 | 2 | 3 | 4 | 5 | 6 |
| 7. | I am good at coordinated movements. | 1 | 2 | 3 | 4 | 5 | 6 |
| 8. | I can perform movements smoothly in most physical activities. | 1 | 2 | 3 | 4 | 5 | 6 |
| 9. | I do physically active things (e.g. jog, dance, bicycle, aerobics, gym, swim) at least three times a week. | 1 | 2 | 3 | 4 | 5 | 6 |
| 10. | I have good sports skills. | 1 | 2 | 3 | 4 | 5 | 6 |
| 11. | Physically, I feel good about myself. | 1 | 2 | 3 | 4 | 5 | 6 |
| 12. | Overall, I am no good. | 1 | 2 | 3 | 4 | 5 | 6 |
| 13. | I find my body handles coordinated movements with ease. | 1 | 2 | 3 | 4 | 5 | 6 |
| 14. | I do lots of sports, dance, gym, or other physical activities. | 1 | 2 | 3 | 4 | 5 | 6 |
| 15. | Most things I do, I do well. | 1 | 2 | 3 | 4 | 5 | 6 |
| 16. | I do sports, exercise, dance or other physical activities almost every day. | 1 | 2 | 3 | 4 | 5 | 6 |
| 17. | I play sports well. | 1 | 2 | 3 | 4 | 5 | 6 |
| 18. | I feel good about who I am physically. | 1 | 2 | 3 | 4 | 5 | 6 |
| 19. | Overall, I have a lot to be proud of. | 1 | 2 | 3 | 4 | 5 | 6 |
| 20. | Nothing I ever do seems to turn out right. | 1 | 2 | 3 | 4 | 5 | 6 |
