# Supplementary material for: Evaluation of the peer leadership for physical literacy intervention: A cluster randomized controlled trial
Source: PLoS One. 2023 Feb 16;18(2):e0280261. doi: 10.1371/journal.pone.0280261 (PMC9934439; doi:10.1371/journal.pone.0280261)
Supplement: S2 File — (PDF) [file pone.0280261.s009.pdf]

ClinicalTrials.gov PRS DRAFT Receipt (Working Version)
Last Update: 04/21/2022 18:21

ClinicalTrials.gov ID: NCT03783767

Study Identification

Unique Protocol ID: H18-00141

Brief Title: Peer Leadership for Physical Literacy (PLPL)

Official Title: Peer Leadership for Physical Literacy: A Randomized Controlled Trial for Elementary School Students

Secondary IDs:

Study Status

Record Verification: April 2022

Overall Status: Completed

Study Start: January 7, 2019 [Actual]

Primary Completion: January 24, 2020 [Actual]

Study Completion: January 24, 2020 [Actual]

Sponsor/Collaborators

Sponsor: University of British Columbia

Responsible Party: Principal Investigator
Investigator: Mark Beauchamp [mbeauchamp_ubc]
Official Title: Professor of Kinesiology
Affiliation: University of British Columbia

Collaborators: Social Sciences and Humanities Research Council of Canada
University of Victoria
Newcastle University

Oversight

U.S. FDA-regulated Drug: No

U.S. FDA-regulated Device: No

U.S. FDA IND/IDE: No

Human Subjects Review: Board Status: Approved
Approval Number: H18-00141
Board Name: Behavioral Research Ethics Board
Board Affiliation: University of British Columbia
Phone: (604) 827-5114
Email: nadia.rad@ors.ubc.ca
Address:

#102, Technology Enterprise Facility III
6190 Agronomy Road
Vancouver, BC V6T 1Z3

Data Monitoring: No

FDA Regulated Intervention: No

Study Description

Brief Summary: The purpose of the proposed research is to develop, implement, and test the efficacy of a theory-driven evidence-based peer leadership program for elementary school students (Grade 6/7; age 11/12 years) in relation to (a) their own leadership skills and their leadership self-efficacy (i.e., confidence to lead), as well as (b) the physical literacy of younger (Grade 3/4; age 8/9) students with whom they are partnered.

Detailed Description: NOTE : Detailed Description has not been entered.

Conditions

Conditions: Leadership
Physical Activity

Keywords: school
intervention
physical literacy
peer mentor
leadership
motor skills
physical activity

Study Design

Study Type: Interventional

Primary Purpose: Prevention

Study Phase: N/A

Interventional Study Model: Parallel Assignment

Number of Arms: 2

Masking: Single (Outcomes Assessor)

Allocation: Randomized

Enrollment: 359 [Actual]

Arms and Interventions

Arms	Assigned Interventions
Experimental: Leadership Intervention Group<br>Teachers in the intervention group will receive a half day workshop from a member of the research team. These teachers will then provide a four week training program to Grade 6/7 students, who will then deliver	Behavioral: Leadership Intervention<br>Grade 6/7 teachers will take part in a workshop on developing transformational leadership among their Grade 6/7 students, and provide teachers with a 4-week training program. Teachers will implement a 4-week leadership development component to their

Arms	Assigned Interventions
a 10-week fundamental movement skill (FMS) training program to Grade 3/4 students.	Grade 6/7 students. Grade 6/7 students will then deliver a 10-week peer-led FMS program to Grade 3/4 students. The Grade 6/7 peer leaders will lead 2 x 30 min sessions per week during physical education classes, recess, or lunch times. In weeks two and three, peer leaders will be observed by members of the research team and provided with feedback using a structured checklist regarding their delivery in the session. Mid-way through the 10-week program teachers will deliver a booster leadership training component to the Grade 6/7 peer-leaders.
No Intervention: Waitlist Control<br>This group of students and teachers will act as a waitlist control group. Therefore, during the same time that the other group is receiving the intervention, this group will proceed with their normal practices.	

Outcome Measures

Primary Outcome Measure:

1. Teacher's rating of their student's transformational leadership using the Transformational Teaching Questionnaire
   Transformational leadership by Grade 6/7 peer leaders will be assessed by teachers using an adaption version of the Transformational Teaching Questionnaire (Beauchamp et al., 2010). This abbreviated measures has been used in our previous studies which demonstrated sound reliability (cronbach alpha > 0.89).

   [Time Frame: Change from Baseline Transformational Leadership at 6 months (Post Intervention)]

Secondary Outcome Measure:

2. Self-Rated Leadership Self-Efficacy using a ten-item questionnaire
   Leadership Self-Efficacy by Grade 6/7 peer leaders will be assessed using a ten-item leadership self-efficacy measure using Bandura's (2006) recommended procedures for assessing self-efficacy.

   [Time Frame: Change from Baseline Leadership Self-Efficacy at 6 months (Post Intervention)]

3. Grade 3/4 student self reported intrinsic motivation using the Intrinsic motivation measure by Sebire and colleagues (2013)
   Intrinsic motivation among Grade 3/4 students will be assessed using the Intrinsic Motivation measure developed by Sebire and colleagues (2013) for specific use with young children in school physical education settings. This measure displays sound reliability (cronbach alpha = 0.77), as well as factorial validity.

   [Time Frame: Change from Baseline Intrinsic Motivation at 6 months (Post Intervention)]

4. Grade 3/4 self-reported Perceived Competence using the Perceived Competence measure developed by Sebire and colleagues (2013)
   Grade 3/4 Perceived Competence will be measured by a questionnaire developed by Sebire and colleagues (2013) for specific use in school physical education settings with young children. This measure has been shown to display sound reliability (cronbach alpha = 0.72).

   [Time Frame: Change from Baseline Intrinsic Motivation at 6 months (Post Intervention)]

5. Physical Self Description Questionnaire (Marsh et al., 2010) will assess Grade 3/4 self-reported self-concept
   The Physical Self Description Questionnaire is a self-report measure for Grade 3/4 students that has sound reliability and factorial validity.

   [Time Frame: Change from Baseline Self-Concept at 6 months (Post Intervention)]

6. Fundamental Movement Skills Competence assessed via the Test of Gross Motor Development (3rd edition) and product scores (throw/kick speed, successful number of catches)
   The kick, throw and a combination throwing/catching test will be performed. Skills will be videoed so that raters can assess the process of each movement and score according to predefined criteria of the Test of Gross Motor

Development 3rd edition (Ulrich and Webster, 2017). Our assessment will also enable us to assess maximal kicking and throwing speed. The combination throwing/catching test will be performed for two trials of 30 seconds each, with scores assigned based on the number of successful catches each participant completes.

[Time Frame: Change from Baseline Fundamental Movement Skills at 6 months (Post Intervention)]

7. School Day Physical Activity will be assessed in Grade 3/4 students using hip worn GT3X+ accelerometers
A subsample of Grade 3/4 students will be randomly selected to wear an accelerometer for one school week (5 days) during the school day only.

[Time Frame: Change from Baseline School Day Physical Activity at 6 Months (Post Intervention)]

Eligibility

Minimum Age: 8 Years

Maximum Age: 14 Years

Sex: All

Gender Based: No

Accepts Healthy Volunteers: Yes

Criteria: Inclusion Criteria:

- Participants must be teachers (Grade 6/7) and students (Grade 3,4, 6 or 7) in elementary schools in the Lower Mainland of British Columbia.
- Students must have a basic working knowledge of English.

Exclusion Criteria:

- There are no exclusion criteria that would exclude anyone who otherwise meets the inclusion criteria specified above

Contacts/Locations

Central Contact Person: Mark R Beauchamp, PhD
Telephone: 604-822-4864
Email: Mark.Beauchamp@ubc.ca

Central Contact Backup: Ryan M Hulteen, PhD
Telephone: 604-822-9140
Email: Ryan.Hulteen@ubc.ca

Study Officials: Mark R Beauchamp, PhD
Study Principal Investigator
University of British Columbia

Locations: **Canada, British Columbia**
Psychology of Exercise, Health, and Physical Activity Laboratory
Vancouver, British Columbia, Canada, V6T 1Z1
Contact: Mark R Beauchamp, PhD 604-822-4864
mark.beauchamp@ubc.ca
Contact: Ryan M Hulteen, PhD 6048229140 ryan.hulteen@ubc.ca

IPDSharing

Plan to Share IPD: Undecided

NOTE : Plan to Share IPD must be 'Yes' or 'No' to satisfy ICMJE policy ('Undecided' is not accepted).

References

Citations:

Nathan N, Sutherland R, Beauchamp MR, Cohen K, Hulteen RM, Babic M, Wolfenden L, Lubans DR. Feasibility and efficacy of the Great Leaders Active StudentS (GLASS) program on children's physical activity and object control skill competency: A non-randomised trial. J Sci Med Sport. 2017 Dec;20(12):1081-1086. doi: 10.1016/j.jsams.2017.04.016. Epub 2017 Apr 21. PubMed 28487140

Beauchamp MR, Barling J, Li Z, Morton KL, Keith SE, Zumbo BD. Development and psychometric properties of the transformational teaching questionnaire. J Health Psychol. 2010 Nov;15(8):1123-34. doi: 10.1177/1359105310364175. Epub 2010 Jun 3. PubMed 20522503

Sebire SJ, Jago R, Fox KR, Edwards MJ, Thompson JL. Testing a self-determination theory model of children's physical activity motivation: a cross-sectional study. Int J Behav Nutr Phys Act. 2013 Sep 26;10:111. doi: 10.1186/1479-5868-10-111. PubMed 24067078

Marsh HW, Martin AJ, Jackson S. Introducing a short version of the physical self description questionnaire: new strategies, short-form evaluative criteria, and applications of factor analyses. J Sport Exerc Psychol. 2010 Aug;32(4):438-82. Erratum in: J Sport Exerc Psychol. 2010 Dec;32(6):909-11. PubMed 20733208

Links:

Available IPD/Information:

Documents

Study Protocol
Document Date: April 23, 2018
Uploaded: 12/19/2018 21:28

U.S. National Library of Medicine | U.S. National Institutes of Health | U.S. Department of Health & Human Services
