# Supplementary material for: Evaluation of the peer leadership for physical literacy intervention: A cluster randomized controlled trial
Source: PLoS One. 2023 Feb 16;18(2):e0280261. doi: 10.1371/journal.pone.0280261 (PMC9934439; doi:10.1371/journal.pone.0280261)
Supplement: S3 File — (PDF) [file pone.0280261.s010.pdf]

Official Title: Peer Leadership for Physical Literacy: A Randomized Controlled Trial for Elementary School Students

Protocol ID: H18-00141

Last Protocol Update To the University of British Columbia Behavioural Research Ethics Board: April 23, 2018

Protocol Submission Date to clinicaltrials.gov: December 19, 2018

RESEARCH PROTOCOL

PARTICIPANTS: Approximately 336 Grade 6/7 students and 1008 Grade 3/4 students from 14 elementary schools in the Lower Mainland of British Columbia, will be invited to participate in this study. The study will involve 1 Grade 6/7 class and 3 Grade 3/4 classes per school. The only inclusion/exclusion criterion involved in this study is that students should have basic working knowledge of English.

RECRUITMENT: Once ethical approval has been obtained from the BREB, school board approval will also be required before recruitment commences. Once BREB and school board approval have been obtained, permission to contact teachers and students will be sought via school principals. Two primary channels will be used to recruit students:

(1) Letters (see attached) will be distributed to parents at least two weeks before scheduled data collection inviting them to consent to their child(ren) to take part in the study.

(2) Verbal announcements will also be made (by a researcher in the students' classes) to students informing them of the study objectives and inviting them to take part in the study. The information conveyed in the verbal announcements will directly align with the information presented in the letter to parents and in the student assent forms.

PARENTAL CONSENT AND STUDENT ASSENT: After ethical approval has been provided by the BREB, we will approach school principals requesting that we contact teachers and students; at this point the researcher will explain the consent procedures to the principals be used in this study. Once school principals and teachers have approved for us to contact students, the information and consent procedures (detailed below) will be explained by the respective research assistant to the students (in their classes). Specifically, students will be briefed about the study orally, with an information letter/consent form sent home to parents (see appended parent consent form). Parents will be required to provide parental consent before their son/daughter can take part in this study. If a parent consents for their son/daughter to take part in the study, students will then be provided with an assent form that they must also sign before they can take part in the study (see appended student assent form). At any point in this study, students can withdraw from the study without experiencing any negative consequences.

REMUNERATION: Physical Activity Kits: Each school (intervention + control schools) will receive two 'Flaghouse Fit-N-Fun Physical Activity Kits' (Developmentally appropriate kits, for children in Grade 3/4, that include physical activity equipment designed to support the delivery of various activities and games designed to enhance physical literacy).

In this study, all children who elect to take part in this study will receive a $10 honorarium for completing pretest and post-test measures. The subsample (n=210) of students who elect to take part in the accelerometry component of the study will receive an additional honorarium for wearing the accelerometers at pre-test and post-test for 5-day blocks (during school hours). Although accelerometers represent a valid and reliable means of collecting physical activity data in free living situations, their use also requires considerate strategies to maximize compliance. With this in mind, participants will be provided with a $20 incentive if they (a) wear the accelerometers at each of the data points, and (b) return the accelerometers.

CONFIDENTIALITY: All participant responses will be de-identified, while also allowing us to connect participants' data across the two data collection windows. Specifically, participants will be asked to provide their names on questionnaires, and in the fundamental movement skills assessments (at pre-and post-test). In addition, Grade 6/7 teachers will rate the leadership behaviors of their students, which will subsequently be linked to students' questionnaires. However, once all the data have been linked (at pre- and post-test) all responses will be de-identified (given a study-specific ID number), with no students' names identifiable within

the electronic database. The document linking participant identifiers with study specific ID codes will be stored in a locked cabinet in the office of the PI (i.e., in a separate room from the de-identified data).

WITHDRAWAL: Participants are free to withdraw from the study at any time without incurring any negative consequences.

FEEDBACK: At the end of the study, a summary report of the findings will be made available to teachers.

TIMELINES: Once institutional and school board approval has been obtained, schools will be stratified and approached to be involved in the study (see attached information letter for principals and teachers – see section 9.6). In order to manage the logistics of the study, the trial will take place in two cohorts (Cohort #1 = 2018/2019; Cohort #2 – 2019/2020). Baseline testing for all students will take place in January (2019 for Cohort 1 and 2020 for Cohort 2). After baseline testing (see attached measures – section 9.5), schools will be randomized to either an intervention condition or a wait-list control condition. In February, teachers of Grade 6/7 students in the intervention condition will be invited to take part in a half workshop as part of one of their one-day professional development days (as per our previous research – see H08-02513). This workshop will centre on how teachers can teach/develop transformational leadership among their Grade 6/7 students, and provide teachers with a 4-week training program that they can subsequently deliver to their Grade 6/7 students in schools.

In February/March those teachers will then implement a 4-week leadership development component to their Grade 6/7 students. Consistent with procedures used in our previous pilot study (Nathan et al, 2017) this 4-week training program (minimum of 4 x 1 hour classes) will involve a peer-leadership training component and a Fundamental Movement Skills (FMS) component. Specifically, Grade 6/7 teachers will provide their students with a practical overview and examples of the four dimensions of transformational leadership, namely idealized influence, inspirational motivation, intellectual stimulation, and individualized consideration. As with our pilot study, this will be delivered in age-appropriate terms (i.e., role modeling, motivating others, helping students to think, and considering others). The FMS component will involve having Grade 6/7 students being taught the correct movement skill patterns for 6 object control skills (catch, underarm throw, overarm throw, kick, two handed strike, and dribble). As per our pilot study, Grade 6/7 peer leaders will be introduced to the structure of a FMS session, to include (1) an introduction to the skill, (2) warm-up game, (3) skill development with key teaching points, (4) skill application to a small-sided game, and 5) cool down and closure. As a guide, Grade 6/7 peer leaders will be encouraged to allocate 2 minutes to the introduction, 5 mins to warm up, 8 mins on skill development, 15 mins on skill application, and 3 mins on cool down and closure. As with our pilot study, Grade 6/7 students will be trained on providing basic teaching cues, individualized feedback and opportunities to practice movement skills in a supportive, fun and encouraging environment. Peer leaders will be provided a whistle, handbook (see section 9.7), which reinforces what they learned in the training. The Grade 6/7 teacher will be provided with a set of laminated 'lesson-plans" that the peer-leaders can use to remind them of the lesson structure. As part of the 4-week training program, peer-leaders will have the opportunity to teach other members of their own (Grade 6/7) short sessions, and receive feedback from their teacher and peers.

Right after the students' spring break (March) those Grade 6/7 students will then deliver a 10-week peer-led FMS program (see section 9.7) to Grade 3/4 students (April-June), followed by post-intervention assessments (mid-June). This 10-week program will directly correspond to the program implemented in our previous pilot study in Australia (see Nathan et al., 2017). Specifically, based on a ratio of 1:3 with eight Grade 6/7 peer leaders per class of 24 Grade 3/4 students (approx. class sizes in BC elementary schools), the Grade 6/7 peer leaders will lead 2 x 30 min sessions per week (spending approximately 3 lessons on each of the object control skills). As with our pilot study in Australia, these

sessions can take place in PE classes, recess, or lunch times (schools will have autonomy in terms of when scheduling these sessions works best for them), and involve (1) an introduction to the FMS, (2) a warm-up game, (3) skill development with key teaching points, (4) skill application to a small-sided game and (5) cool-down and closure. At the end of the first session members of the research team (research assistants) will debrief with the peer leaders to discuss any challenges, concerns and problem solve ideas for running the physical activity session with their groups. In weeks two and three, peer leaders will be observed by members of the research team (research assistants) and provided with feedback using a structured checklist regarding their delivery session (see section 9.5). Mid-way through the 10-week program (week 5) teachers will deliver a booster leadership training component to the Grade 6/7 peer-leaders. At the end of the 10-week FMS program (June), post-test assessments will take place (see section 9.5).

Students in schools in the wait-list control condition will complete the same post-test measures as those in the intervention condition (1 Grade 6/7 class and 3 Grade 3/4 classes per school), but without receiving any of the intervention materials. The control schools will be asked to follow usual practice during the study period. Control schools will be offered the same leadership training and intervention materials in the subsequent year.

MEASURES

Questionnaires: *Transformational Leadership* by Grade 6/7 peer leaders will be assessed by teachers using an adapted and shortened version of the Transformational Teaching Questionnaire (TTQ). We used this abbreviated measure in our previous pilot study, which demonstrated sound reliability ($\alpha \geq .89$). ***Leadership Self-efficacy*** by Grade 6/7 peer leaders will be assessed using a ten-item leadership self-efficacy measure, using Bandura's (2006) recommended procedures for assessing self-efficacy. ***Intrinsic Motivation*** among Grade 3/4 students (aged 8/9 years) will be assessed using the Intrinsic Motivation measure developed by Sebire and colleagues (2013) for specific use with young children (aged 7-11) in school physical education settings. This measure was found to display sound reliability ($\alpha = .77$), as well as factorial validity in relation to the other motivation regulations subsumed within self-determination theory. **Perceived competence** among Grade 3/4 students will be assessed using the competence measure developed by Sebire and colleagues (2013) for specific use with young children (aged 7-11) in school physical education settings. This measure was found to display sound reliability ($\alpha = .72$). **Self-concept.** Physical self-concept among Grade 3/4 students will be assessed using the short version of Physical Self Description Questionnaire developed by Marsh et al. (2010). This instrument has been found to demonstrate sound reliability and factorial validity.

Fundament al Movement Skills among Grade 3/4 students will be assessed using the Test of Gross Motor Development-3 (TGMD-3; Ulrich, 2000). Consistent with procedures used in our pilot study, participants will be videotaped performing two trials of the six fundamental skills. Research assistants, blinded to group allocation, will assess the videos according to behavioural components. Each skill component is scored a "1" if observable and performed correctly; if they perform it incorrectly the component is given a score of "0". This procedure will be completed for each of the two trials, with trial scores summed to calculate a total score for each skill. An overall object control skill score is calculated by summing the skill scores.

Within School Physical Activity: a subsample of Grade 3/4 students (n = 210) will be randomly selected and invited to wear a small physical activity monitor (accelerometer) on two occasions; at baseline and post-test for one school week (5-days at each data point). Specifically, of those who indicate an interest in wearing the accelerometers, 5 students per class in both the intervention and control condition (3 classes x 5 students per class x 14 schools = 210) will be invited to wear the accelerometer.

Accelerometers provide a valid, reliable, and objective method of collecting physical activity data within free-living physical activity settings, and have been demonstrated to be feasible for use with children. An accelerometer is the size of a USB Memory Stick and is worn around the participant's waist. Not only do accelerometers enable researchers to identify the amount of energy expenditure on a daily basis (typically accelerometer studies involve one week assessment protocols), but they also provide invaluable insight into when (in any given day) physical activity is pursued. From the perspective of this research program, accelerometers will enable us to examine the extent to which children engage in physical activity within school hours (as measured by minutes of moderate-to-vigorous physical activity: MVPA), and how changes in these behaviours are predicted by class assignment to the intervention condition. The research team has considerable experience with using accelerometers with youth in research settings.
